# Investigating the degradation potential of microbial consortia for perfluorooctane sulfonate through a functional "top-down" screening approach

**DOI:** 10.1371/journal.pone.0303904

**Published:** 2024-05-17

**Authors:** Yu Liang, Anzhou Ma

**Affiliations:** 1 Research Center for Eco-Environmental Sciences, Chinese Academy of Sciences, Beijing, China; 2 University of the Chinese Academy of Sciences, Beijing, China; Universidade Estadual de Ponta Grossa, BRAZIL

## Abstract

Perfluorooctane sulfonate (PFOS) is a prominent perfluorinated compound commonly found in the environment, known to pose various risks to human health. However, the removal of PFOS presents significant challenges, primarily due to the limited discovery of bacteria capable of effectively degrading PFOS. Moreover, single degradation bacteria often encounter obstacles in individual cultivation and the breakdown of complex pollutants. In contrast, microbial consortia have shown promise in pollutant degradation. This study employed a continuous enrichment method, combined with multiple co-metabolic substrates, to investigate a microbial consortium with the potential for PFOS degradation. By employing this methodology, we effectively identified a microbial consortium that demonstrated the capacity to reduce PFOS when exposed to an optimal concentration of methanol. The consortium predominantly comprised of Hyphomicrobium species (46.7%) along with unclassified microorganisms (53.0%). Over a duration of 20 days, the PFOS concentration exhibited a notable decrease of 56.7% in comparison to the initial level, while considering the exclusion of adsorption effects. Furthermore, by comparing the predicted metabolic pathways of the microbial consortium with the genome of a known chloromethane-degrading bacterium, Hyphomicrobium sp. MC1, using the KEGG database, we observed distinct variations in the metabolic pathways, suggesting the potential role of the unclassified microorganisms. These findings underscore the potential effectiveness of a "top-down" functional microbial screening approach in the degradation of stubborn pollutants.

## 1. Introduction

Perfluorinated compounds (PFCs) are a class of compounds where all hydrogen atoms in hydrocarbon compounds and their derivatives are replaced by fluorine atoms [1–3]. Among these compounds, perfluorooctane sulfonate (PFOS) is a common example found in the environment and serves as the ultimate transformation product of numerous other PFCs [4–7]. PFOS poses various risks to biological health, and studies have linked it to the incidence of human bladder cancer [8]. Additionally, PFOS has been detected in animals, human blood, dust, and water bodies [9–11].

Despite the significant risks associated with PFOS, its removal remains a challenging task [12–14]. Common methods used for PFOS removal include physical separation and chemical degradation, both of which are expensive and complex [15,16]. Bioremediation, a simple and cost-effective remediation method, has been employed for the treatment of various pollutants. However, the bioavailability of PFOS is severely limited due to factors such as the high bond energy of C-F bonds within PFOS and the protective shell formed by fluorine atoms [17]. As a result, only a few PFOS-degrading bacteria have been reported thus far. For instance, Huang and Jaffe [18] discovered Acidimicrobium sp. A6, which is capable of partially degrading PFOS under anaerobic conditions.

However, the degradation of pollutants by individual species presents a range of challenges in the field. Current research suggests that a single species may lack certain genes or enzymes in their metabolic pathways, leading to the accumulation of toxic byproducts and incomplete degradation of pollutants [19]. Moreover, individual species are vulnerable to environmental disturbances that may impact their functional capabilities [20]. Despite the discovery of numerous bacteria with degradation capabilities, many of them remain uncultivable. Molecular biology analyses have revealed unexplored functionalities in these uncultured microorganisms. Mei et al. [21] demonstrated the crucial role of an uncultivated bacterium in amino acid degradation through transcriptomic and metagenomic analyses. There are several reasons for the uncultivability of microorganisms, including microbial interactions. Some microorganisms engaged in symbiotic relationships are unable to survive independently when separated from other members [22]. All of these factors impose limitations on the utilization of microbial resources.

Synthetic microbial consortia pertain to microbial communities consisting of two or more genetically modified cell populations or artificially constructed community systems achieved by co-cultivating multiple species under controlled environmental conditions [23,24]. Unlike single species, which encounter numerous obstacles, synthetic microbial consortia aim to mimic natural microbial communities. Notably, synthetic microbial consortia have exhibited enhanced efficiency in degrading specific substances when compared to individual species [25]. For example, Rodríguez-Castillo et al. [26] successfully enriched a nicotine-degrading consortium from agricultural soil, consisting of eight bacterial strains and one yeast strain, which collectively degraded approximately 95.8% and 94.4% of distinct nicotine combinations. Additionally, for recalcitrant or high-concentration pollutants, the addition of auxiliary substances such as simple carbon sources can stimulate degradation. These substances provide nutrients, enhance pollutant bioavailability, and accelerate microbial degradation processes [27]. Synthetic microbial consortia can uncover additional microbial functionalities, offering innovative strategies for the biodegradation of recalcitrant pollutants [28–31]. Previous studies have provided evidence of researchers reporting their findings across diverse scientific disciplines in order to advance knowledge. Within these scientific reports, particular attention has been directed by many researchers towards fields associated with medicine, bacteria, and pollution absorption [32–35].

Despite persistent efforts, the biodegradation of PFOS remains a significant challenge due to its recalcitrant nature and the limited discovery of effective PFOS-degrading bacteria. Moreover, relying solely on individual bacterial species for PFOS degradation presents drawbacks, including incomplete degradation due to missing genes or enzymes and instability in the face of environmental disturbances. While synthetic microbial consortia hold promise for pollutant degradation by mimicking natural microbial communities, research on their application to PFOS degradation is limited. Therefore, the objective of this study was to employ a "top-down" functional screening approach using synthetic microbial consortia to identify novel capabilities for PFOS degradation. Soil samples contaminated with PFOS over a long period were subjected to extended enrichment and acclimatization in the laboratory under various conditions, including the presence of auxiliary substrates and PFOS stress. The objective of this study was to investigate and resolve lingering inquiries regarding the degradation capabilities of microbial consortia towards persistent pollutants like PFOS. By identifying the microbial community structures that displayed PFOS reduction, valuable insights into the constituent microorganisms and their metabolic interactions were obtained. To emulate natural attenuation processes and unveil previously uncultivated degrading functions, contaminated environmental samples were utilized in conjunction with a selective enrichment method. This study built upon previous efforts by applying a synthetic ecology approach to tackle the degradation of PFOS, a highly persistent pollutant. By elucidating microbial consortia with PFOS degradation capabilities, innovative bioremediation strategies can be developed, and the performance of these consortia can be optimized through consortium design.

## 2. Materials and methods

### 2.1 Chemicals and media

PFOS-K, characterized by the chemical formula C_8_F_17_KO_3_S, was employed as a substitute for PFOS in our experiments due to its low solubility in water. A mineral salt medium (MSM) was employed, composed of the following components per liter: NaNO_3_ (0.5 g), KH_2_PO_4_ (1.0 g), CaCl_2_ (0.02 g), MgSO_4_ (0.2 g), (NH_4_)_2_SO_4_ (0.5 g), NaH_2_PO_4_·H_2_O (1.0 g), FeSO_4_·7H_2_O (0.001 g), and 2 mL of trace element solution. The trace element solution was prepared as follows per liter: MoO_3_ (0.001 g), ZnSO_4_·7H_2_O (0.007 g), CuSO_4_·5H_2_O (0.0005 g), H_3_BO_3_ (0.001 g), MnSO_4_·5H_2_O (0.001 g), CoCl_2_·6H_2_O (0.001 g), NiSO_4_·7H_2_O (0.001 g). Additionally, 0.1345 g of PFOS-K was added per liter of medium to achieve a PFOS concentration of 0.25 mmol·L^−1^. The medium was sterilized at 121°C for 20 minutes. To enhance microbial utilization of PFOS and its salts, co-metabolic substrates including glucose, n-octane, and methanol were included in the media, maintaining a consistent carbon content of 0.25 mmol·L^−1^. All chemicals and solvents used were of analytical grade.

Soil samples were collected in the vicinity of a PFOS production facility in Wuhan, China. Surface soil was obtained from multiple locations and thoroughly mixed. After passing through a 2 mm soil sieve, it was stored at 4°C.

### 2.2 Acquisition of microbial consortia

To obtain microbial consortia, 2 g of soil samples were added to the medium and cultivated in a shaker at 30°C and 150 r/min for 20 days. Then, 2 mL of the culture was transferred to the same medium. This process was repeated for three successive transfers, and the PFOS-K concentration was assessed at the end of each transfer cycle. The initial screening stage continued until the first occurrence of decreased PFOS-K concentration. No replicates were conducted during the initial screening stage. Following the initial screening stage, three sets of replicates were established for all media.

### 2.3 PFOS concentration

**Fig 1:**
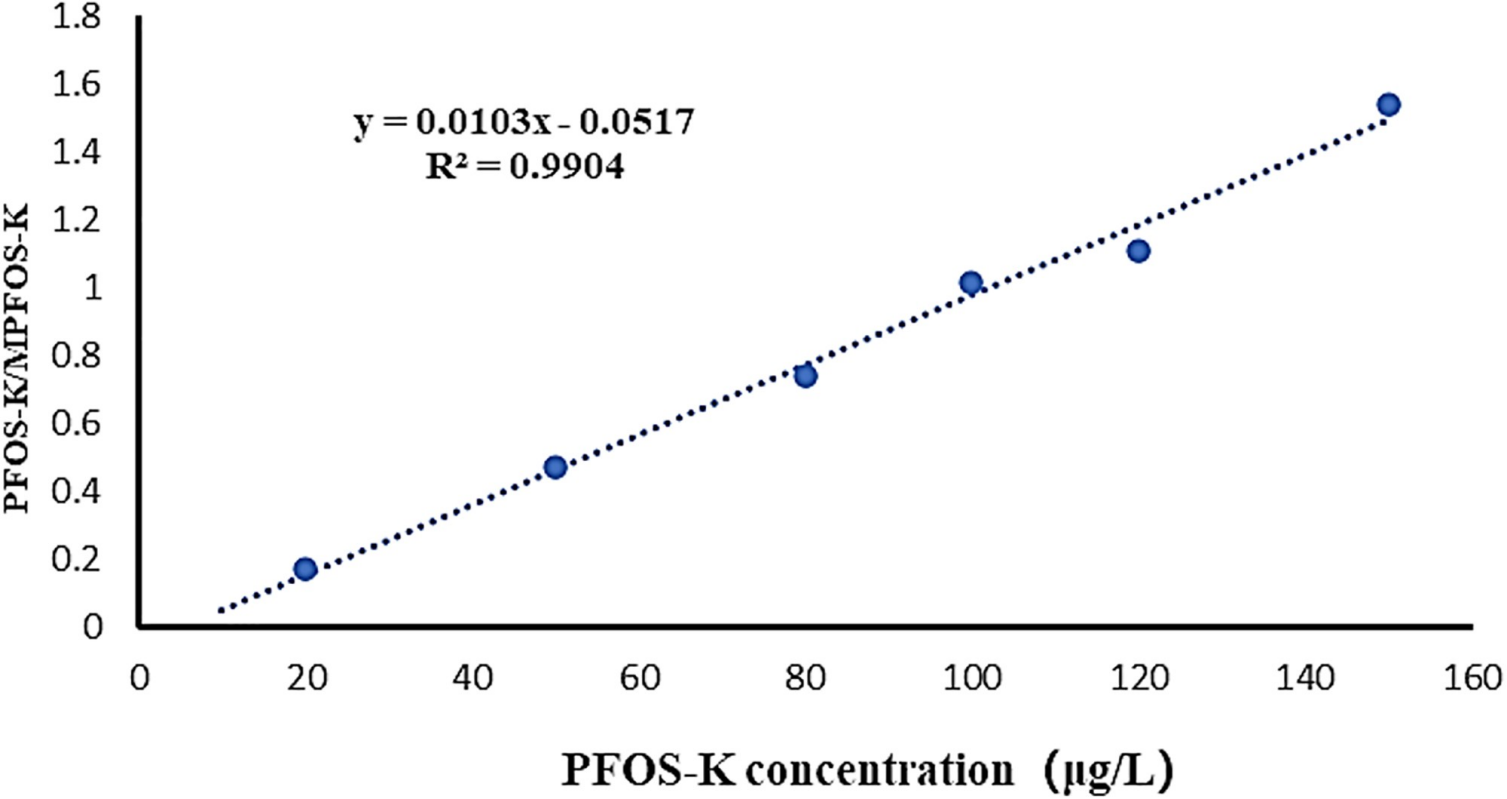
Generation of the standard curve using the PFOS-K detection method implemented in the experiment.

The pre-treatment of PFOS-K in the culture medium followed the methodology described in the study by Huang and Jaffe [18]. PFOS-K in the culture medium was diluted approximately 1000 times with methanol. Subsequently, the samples were filtered through a 0.22 μm organic phase filter and injected into sample vials for analysis. A standard curve for PFOS-K quantification was established within the range of 10–150 μg·L^−1^. In both the samples and the standard curve, 50 μg·L^−1^ of MPFOS-K was added as an internal standard. Finally, the response ratio of PFOS-K/MPFOS-K was used as the vertical coordinate, and the concentration of PFOS-K was used as the horizontal coordinate to construct the standard curve (Fig 1).

UPLC-MS (Waters ACQUITY UPLC H-Class PLUS & Xevo TQ-S Micro) was employed for the detection of PFOS-K. The chromatographic column utilized was a Waters BEH C18 column, with mobile phase A consisting of a 5 mM aqueous solution of acetic acid, and mobile phase B consisting of a methanol solution, at a flow rate of 0.2 mL/min. A gradient elution program was employed (see Table 1). The mass spectrometry parameters were as follows: An ESI ion source was operated in negative ion mode with multiple reaction monitoring (MRM) as the scanning method. The capillary temperature was set at 350°C, the spray voltage at 3000 eV, the ion source temperature at 200°C, and the column oven temperature at 30°C. The parent ion was 498.9, the daughter ion was 80.2, and the collision energy was 55 eV.

**Table 1:** Gradient elution program for PFOS-K analysis.

Time (min)	A%	B%
0	90	10
6	35	65
7	25	75
11	0	100
13	0	100
17	90	10

### 2.4 DNA extraction and sequencing analysis

Following each generation of cultivation, 2 mL of the sample was collected for DNA extraction. The extraction procedure was conducted according to the instructions provided in the bacterial whole-genome DNA extraction kit (TIANGEN TIANamp Bacteria DNA kit-DP302). The V3-V4 region of the bacterial 16S rRNA gene was targeted for amplification using primers 338F (ACTCCTACGGGAGGCAGCA) and 806R (GGACTACHVGGGTWTCTAAT). The PCR reaction system consisted of 25 μL of 2x Premix Taq, 1 μL of the forward primer, 1 μL of the reverse primer, and 50 ng of template DNA. Nuclease-free water was added to reach a final volume of 50 μL. PCR amplification was performed using the BioRad S1000 system (Bio-Rad Laboratory, CA). The resulting PCR products were analyzed through 1% agarose gel electrophoresis. Subsequently, the amplified products were sent to Shenzhen MAGIGENE Co., Ltd. (China) for sequencing.

### 2.5 Statistics and analysis

The raw data obtained from sequencing underwent processing and modification using Fastp (Release 0.19.6, OpenGene/fastp) and FLASH (Release 1.2.11, https://ccb.jhu.edu/software/FLASH/index.shtml) software. Subsequently, the sequences were processed utilizing QIIME, followed by OTU clustering and statistical analysis performed using Uparse and Usearch (Release 7, http://www.drive5.com/usearch/). To predict the KEGG and COG functions of the bacterial 16S sequences, PICRUSt (Release 1.1.0, http://picrust.github.io/picrust/) software was employed.

## 3. Results and discussion

### 3.1 PFOS-K concentration

The soil samples utilized in this study were acquired from the vicinity of a PFOS production facility in China, aiming to capture microbial communities that might have been exposed to and acclimated to PFOS contamination over a prolonged period. Prior to their utilization in the experiments, these soil samples underwent thorough mixing and were sieved through a 2 mm mesh.

The microbial consortia were obtained through the addition of 2 g of the soil samples to a MSM containing PFOS-K, followed by incubation in a shaker at 30°C and 150 r/min for a duration of 20 days. This initial enrichment step was repeated thrice, with a 2 mL aliquot of the culture transferred to fresh MSM medium after each 20-day incubation period. Monitoring of the PFOS-K concentration occurred at the conclusion of each transfer cycle, enabling the tracking of alterations in PFOS-K levels. The designation WH4 corresponds to the microbial consortium acquired through this enrichment process, which exhibited a substantial reduction in PFOS-K concentration when methanol was introduced as a co-metabolic substrate during the initial screening.

**Fig 2:**
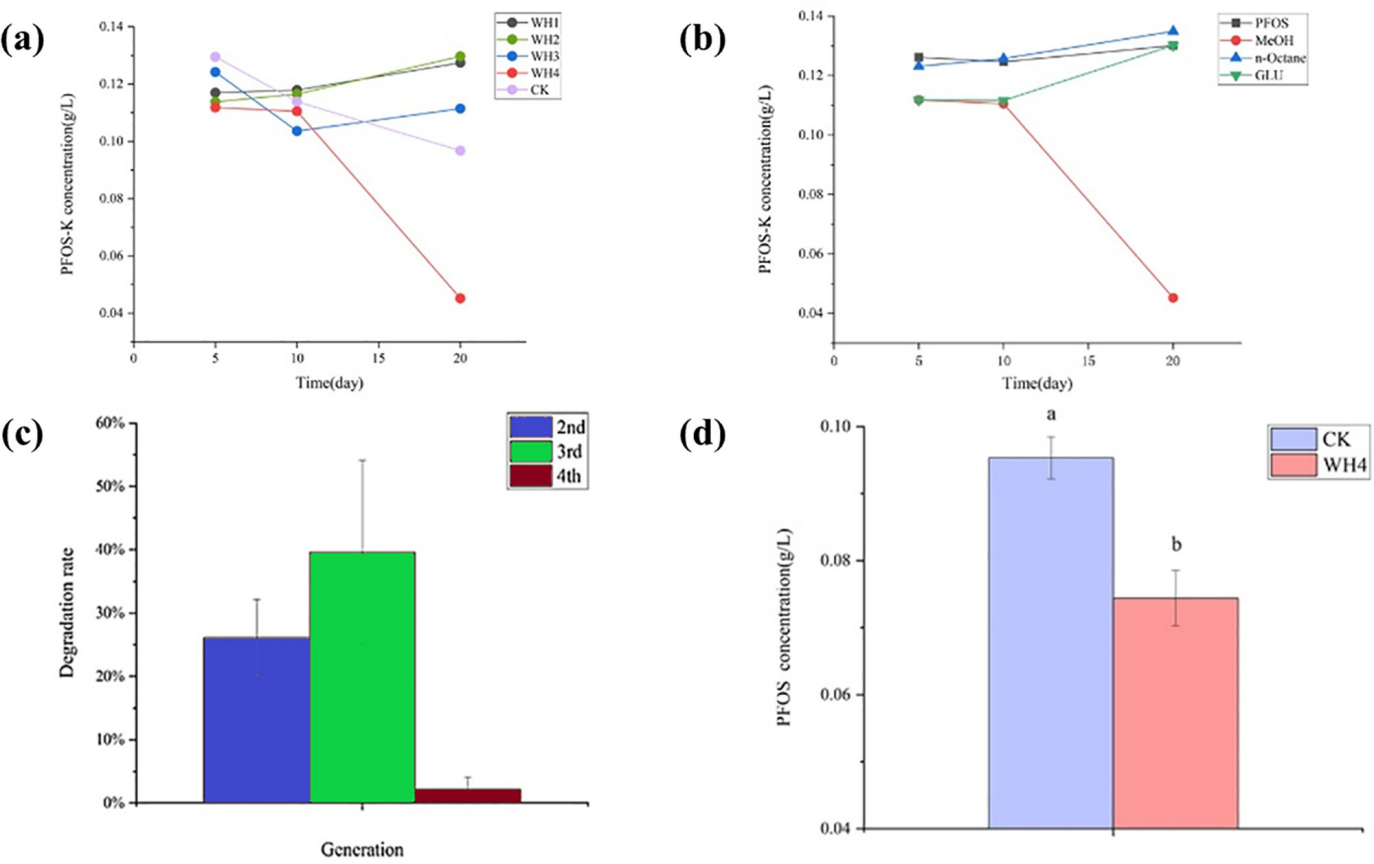
Changes in PFOS-K concentration during the preliminary screening process. (a) Concentration changes of PFOS-K in different samples with methanol as a co-metabolite substrate. (b) PFOS-K concentration changes of WH4 samples under different co-metabolized substrates. (c) Changes in the degradation rate of PFOS-K from the second to the fourth generation. (d) PFOS-K concentration in the supernatants of the second generation WH4 and CK samples.

Fig 2(a) demonstrates that, during the initial screening stage, a 53.3% decrease in PFOS-K concentration in the WH4 consortium on the 20th day was observed when methanol was introduced as a co-metabolic substrate. This decrease was compared to the control group, which did not receive any co-metabolite. In contrast, Fig 2(b) reveals that when the WH4 consortium was exposed to other co-metabolized substrates such as glucose or n-hexane, or when no co-metabolite was provided, no significant reduction in PFOS-K concentration was observed over the same time period. Hence, it can be inferred that the presence of methanol specifically enhanced the PFOS-K degradation capabilities of the WH4 consortium, particularly during the initial screening stage.

A control experiment was conducted to investigate the potential impact of methanol on PFOS-K concentration in the absence of bacteria. The control experiment involved utilizing the same MSM supplemented with PFOS-K and various co-metabolic substrates (glucose, n-octane, and methanol), without the inclusion of any soil sample or microbial inoculum. Throughout the abiotic control incubations, the PFOS-K concentration remained stable. This stability suggests that the observed decrease in PFOS-K levels within the WH4 consortium was primarily attributed to the metabolic activities of the microbial community, rather than being solely a result of chemical interactions with the added substrates.

Furthermore, WH4 was passaged using MSM medium containing PFOS-K, with methanol employed as a co-metabolic substrate. The phenomenon of reduced PFOS-K concentration was observed in both the second and third generations, with degradation rates of 26.1% and 39.6%, respectively (Fig 2c). However, as the number of transmissions increased, the error gradually increased, and the phenomenon disappeared in the fourth generation. To eliminate the influence of adsorption, we centrifuged the bacterial solution from the second generation and examined the supernatant to determine the concentration of PFOS-K. It was observed that the concentration of PFOS-K was significantly reduced compared to the control group, thus ruling out the influence of adsorption (Fig 2d).

In this experiment, a decrease in PFOS-K concentration in the microbial consortium was observed when methanol was added as a co-metabolite. PFOS, being a recalcitrant pollutant, poses a challenge for microorganisms to metabolize due to the limited availability of carbon and energy sources [36]. However, the addition of methanol can provide microorganisms with the necessary energy and carbon sources, thereby promoting their growth and metabolic activity. Furthermore, methanol can enhance the activity of relevant enzymes in microbial metabolic pathways, thereby accelerating the reaction rate of key processes and improving degradation efficiency.

Previous research by Guo et al. [37] demonstrated that the addition of methanol facilitated the debromination process of 2,2’,4,4’-tetrabromodiphenyl ether (BDE-47). Methanol has been found to promote pollutant degradation in various cases. For instance, it has been shown to reduce the toxicity of coal chemical wastewater and enhance the degradation rate of phenolic compounds. Shi et al. [38] utilized methanol to enhance the biodegradation of phenolic and NHCs in coal pyrolysis wastewater.

**Fig 3:**
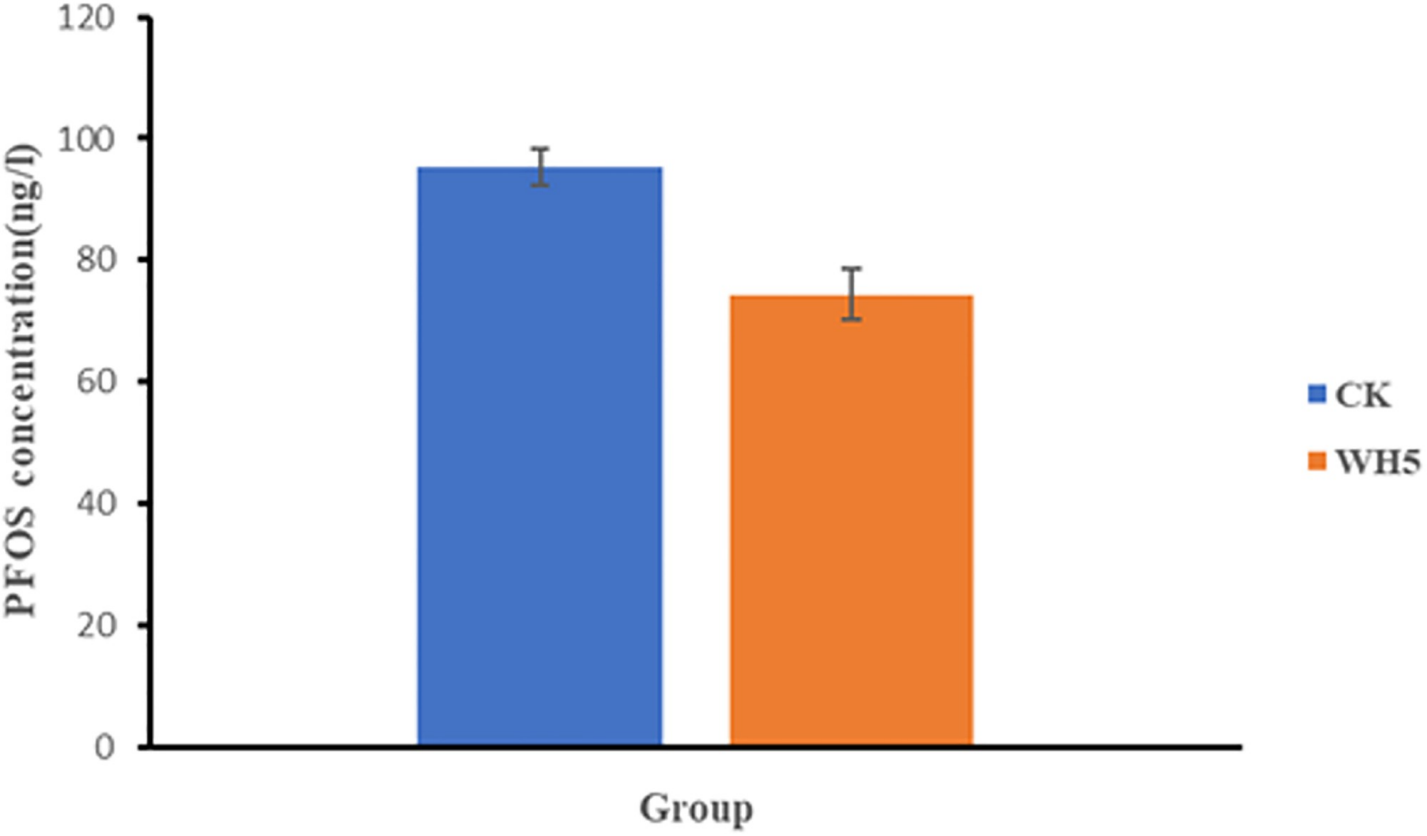
PFOS-K concentration comparison between centrifuged supernatants of second-generation WH4 and control cultures.

The influence of adsorption was investigated by analyzing the PFOS-K concentration in the supernatant of the second-generation WH4 culture compared to the control (Fig 3). The supernatant derived from the second-generation bacterial solution of WH4, obtained through centrifugation, exhibited a markedly lower PFOS-K concentration compared to the control supernatant without centrifugation. This finding effectively ruled out the impact of adsorption and confirmed the genuine degradation of PFOS-K within the WH4 consortium at this particular stage.

**Fig 4:**
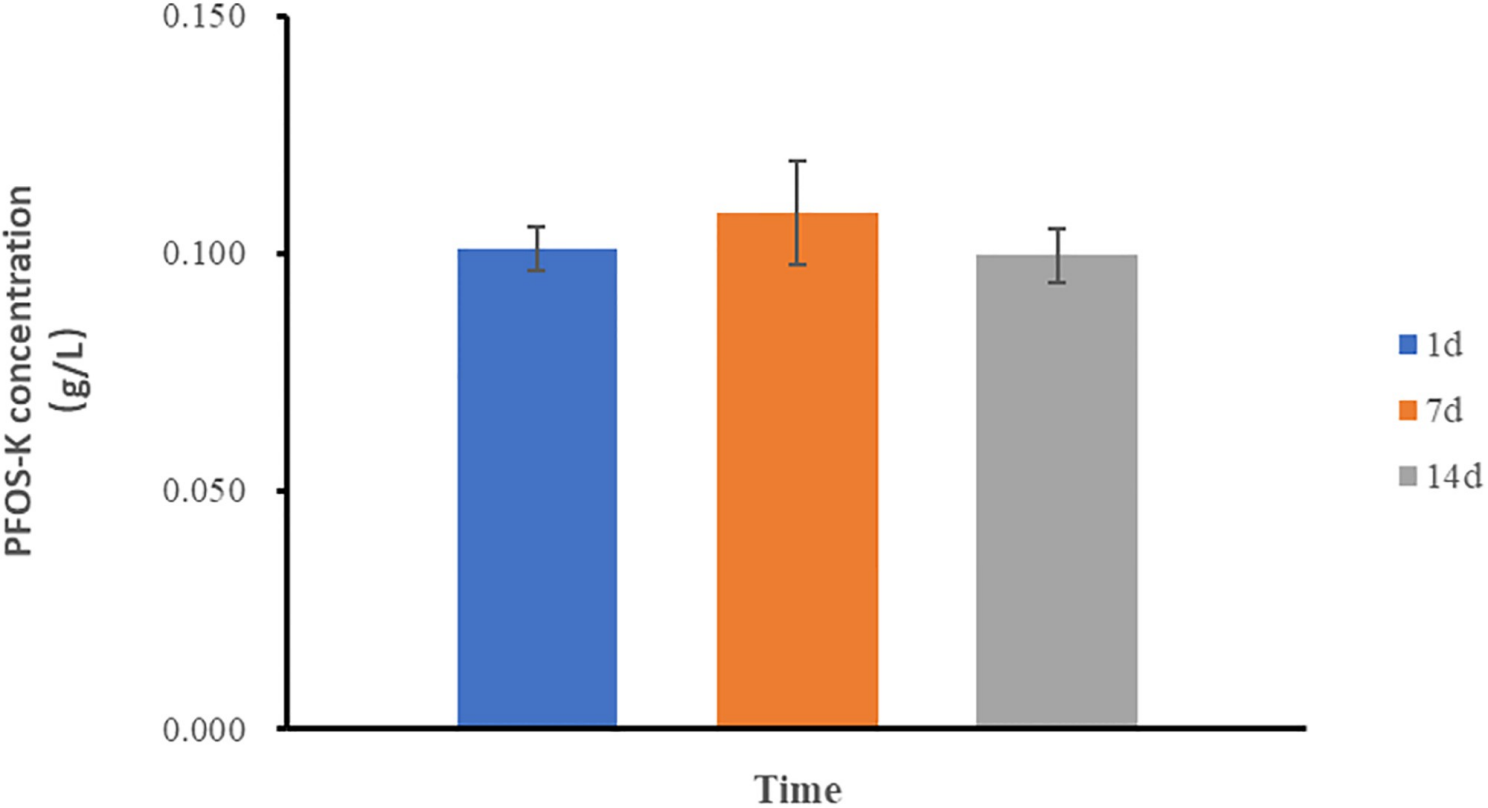
Concentration variation of PFOS-K in the third generation of the WH4.

In order to examine the long-term sustainability of the observed phenomenon of PFOS-K reduction in the WH4 consortium, the concentration of PFOS-K was specifically monitored during the third generation (Fig 4). The results revealed a persistent downward trend in PFOS-K concentration until the third generation, indicating continuous reduction of PFOS-K. However, the degradation rate displayed a fluctuating pattern, and the error bars noticeably widened, suggesting a destabilization of the phenomenon with further subculturing. When considering these findings alongside the previous observations, it can be inferred that the presence of methanol as the auxiliary substrate may have ultimately hindered the long-term PFOS degradation capability of the consortium during serial transfer.

**Fig 5:**
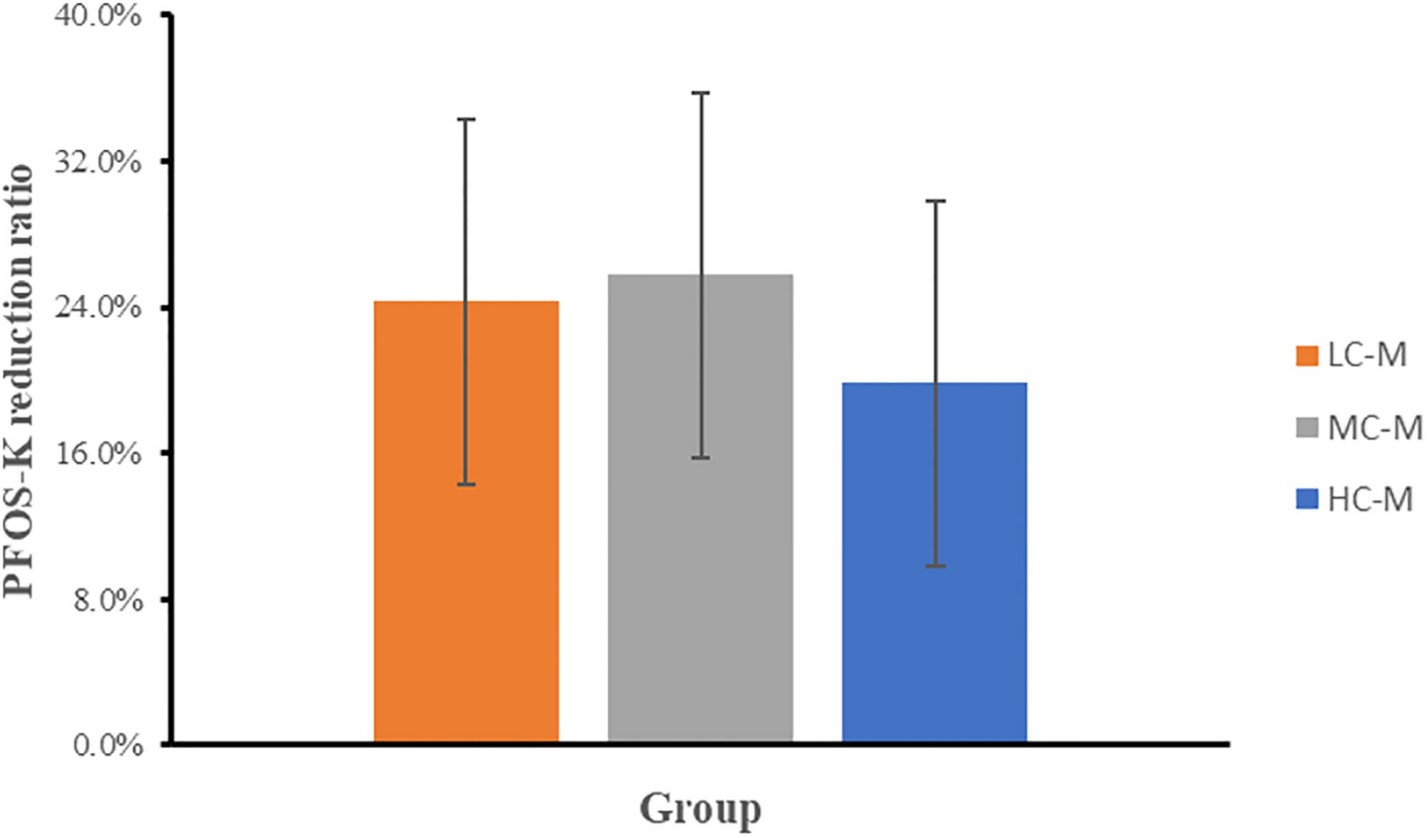
PFOS-K reduction rate in WH4 under different methanol concentrations. MC-M corresponds to the initial screening conditions (equivalent to 0.25 mmol/L C), LC-M denotes one-fifth of MC-M, and HC-M represents twice the concentration of MC-M.

Regrettably, the reduction of PFOS-K cannot be sustained as the number of generations increases. The analysis of the degradation rate reveals an increasing standard deviation, indicating a loss of stability in the phenomenon. Eventually, by the third generation, the phenomenon disappears. It is hypothesized that the coexistence of methanol may contribute to the unsustainability of this phenomenon. The influence of methanol concentration on PFOS-K degradation was investigated in first-generation samples (Fig 5). It was observed that the degradation of PFOS-K was affected by the amount of methanol added, with higher methanol concentrations resulting in a decrease in the PFOS-K degradation rate. Organisms possess various mechanisms to acquire new metabolic pathways [39]. However, when pathways or processes serve the same function, degeneration can occur, as seen in the case of the multiple alternative pathways for glucose metabolism [40]. Methanol is more readily utilized compared to PFOS-K, and the ability to degrade PFOS-K may gradually diminish with successive subculturing.

### 3.2 Microbial consortium structure

**Fig 6:**
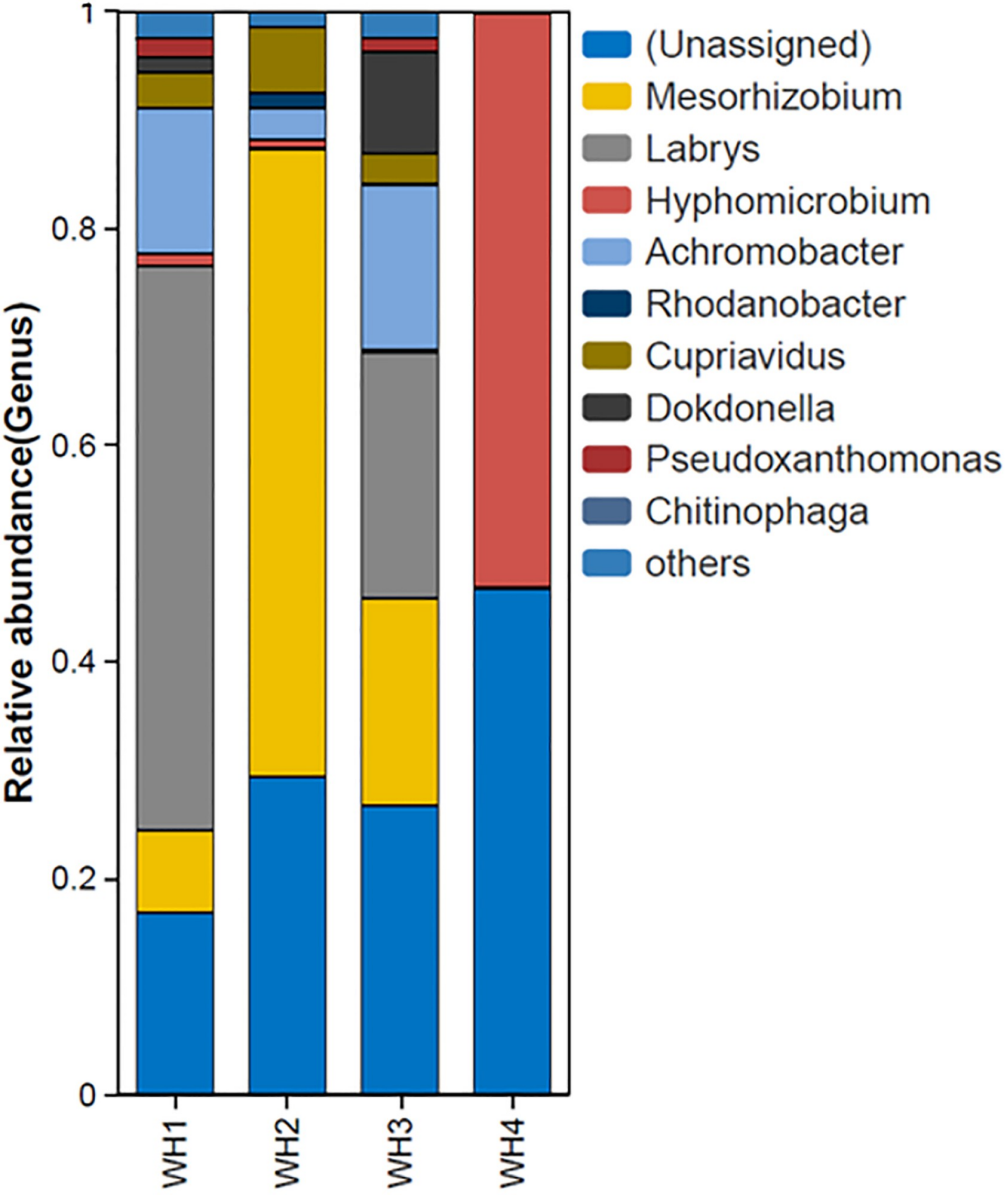
Microbial consortium structure using methanol as a co-metabolic substrate.

A relative abundance analysis at the genus level was conducted for the first generation treatment using methanol as a co-metabolic substrate. The results revealed that the WH4 group, which exhibited a reduction in PFOS-K concentration, primarily consisted of two species: Hyphomicrobium (46.7%) and unclassified microorganisms (53.0%). Although Hyphomicrobium was also present in other samples, its proportion was significantly lower, such as 1.1% in WH4, whereas other samples exhibited a higher number of species compared to WH4. Therefore, the microbial consortium structure of WH4 was distinct from other groups, with the key difference being the presence of Hyphomicrobium (see Fig 6).

The microbial community composition within the WH4 consortium was analyzed, revealing its primary composition of two dominant taxonomic groups: Hyphomicrobium, responsible for 46.7% of the total community, and a group of unclassified microorganisms, representing 53.0% of the consortium. The most abundant genus identified was Hyphomicrobium, while the remaining 53.0% consisted of multiple unclassified operational taxonomic units (OTUs) or amplicon sequence variants (ASVs) that could not be confidently assigned to known taxa at the species or genus level.

This composition suggests that the WH4 consortium, which exhibited a notable reduction in PFOS-K concentration, was not solely a binary culture composed of Hyphomicrobium and one other unclassified species. Instead, it harbored a diverse but uncharacterized microbial community, with Hyphomicrobium acting as the predominant and potentially key player in the observed PFOS-K degradation, while the unclassified members likely contributed complementary metabolic capabilities or provided supportive roles. The challenges associated with working with complex consortia containing uncultured microorganisms are underscored by the inability to isolate and functionally validate the Hyphomicrobium strain, as mentioned in the manuscript.

Previous research has indicated that Hyphomicrobium harbors dehalogenase enzymes capable of degrading various halogenated compounds. For instance, Sun et al. [41] identified a microbial community with the ability to degrade halogenated polycyclic aromatic hydrocarbons (HPAH), where the genus Hyphomicrobium demonstrated efficient degradation of 9-BrAnt, 2-BrPhe, and 2-ClPhe. Moreover, the dehalogenase gene dcm in Hyphomicrobium exhibited some genetic variability. Hayoun et al. [42] discovered that Hyphomicrobium sp. MC8b could degrade dichloromethane (DCM) and identified a distinct set of genes through pan-proteomic analysis, separate from the traditional dcm gene. PFOS, being a halogenated compound, shares certain similarities with DCM. It is highly plausible that the Hyphomicrobium present in our acquired microbial consortium possesses some capability for PFOS degradation. Unfortunately, isolating this strain for functional validation was not feasible. Additionally, there were several unclassified microorganisms in the consortium, which may have contributed to the degradation process of Hyphomicrobium or played a supportive role. Therefore, these microorganisms formed a consortium through environmental filtering, but their functions were not stable and eventually diminished.

To comprehend the potential shifts in the microbial community structure that might have contributed to the observed decline in PFOS-K degradation in later generations of the WH4 consortium, an analysis of the bacterial community composition was conducted on the 4th generation samples. The results unveiled a noteworthy alteration in the relative abundances of dominant taxa when compared to earlier generations.

In the 4th generation, there was a significant decrease in the proportion of Hyphomicrobium, which had initially been the predominant genus in the WH4 consortium, accounting for only 12.4% of the total community. Simultaneously, the relative abundance of unclassified microorganisms also exhibited a decline, reaching 29.6%. Conversely, other genera, such as Mycobacterium (18.2%) and Sphingomonas (16.5%), became more prevalent in the 4th generation samples. This shift in the microbial community structure suggests that the long-term stability and PFOS-K degradation capability of the WH4 consortium were compromised over successive subculturing, potentially attributable to the decreasing dominance of the key Hyphomicrobium and unclassified populations.

To shed further light on the relationship between methanol availability and the microbial community structure, additional experiments were conducted to monitor changes in the consortium composition under varying methanol concentrations, with the potential influence of PFOS-K excluded. The observed results demonstrated that higher methanol concentrations (HC-M) elicited a relative increase in the abundance of taxa known to utilize methane or methanol as carbon sources, notably Methylobacterium and Methylophilus. In contrast, lower methanol levels (LC-M) fostered the proliferation of microorganisms possessing a more diverse metabolic repertoire, including Hyphomicrobium and other unclassified members. These findings suggest that maintaining a balance between methanol-utilizing organisms and versatile degraders within the consortium may emerge as a critical factor in preserving the long-term PFOS-K degradation capabilities. The preferential utilization of methanol may potentially outcompete the PFOS-K degradation pathways over successive generations.

### 3.3 Predicting the potential function of the microbial consortium

**Fig 7:**
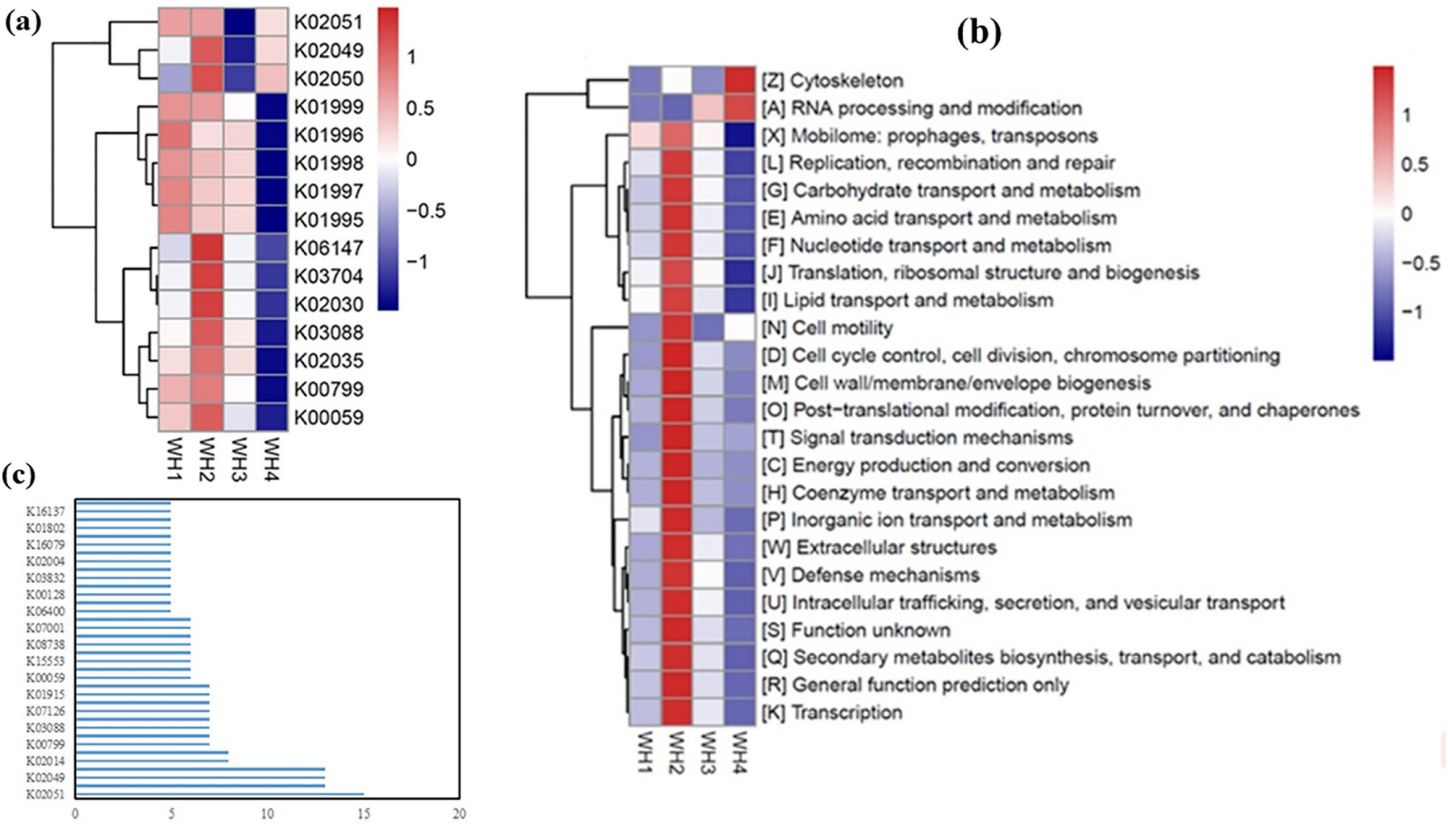
Functional prediction of 16S rRNA genes based on (a) KEGG and (b) COG annotations. (c) KEGG enrichment analysis chart for Hyphomicrobium sp. Strain MC1.

Insights into potential metabolic differences between the WH4 consortium and other sample groups were obtained through a comparative functional prediction analysis based on 16S rRNA gene sequences. A heatmap presenting the top 15 orthologous KEGG pathways (k0-level) identified across distinct treatment points was generated using Silva taxonomy, Tax4Fun, and KEGG annotation of 16S data (Fig 7a). A lower relative abundance of most pathways was observed in WH4 compared to other samples, with only k02051, k02049, and k02050 showing higher values, aligning with their roles in amino acid transport. Furthermore, the 25 most abundant COG functional categories present across samples were identified (Fig 7b), revealing a lower abundance of 23 dominant groups in WH4 and an elevated representation of RNA/cell functions. This pattern likely arises from the effects of PFOS toxicity on core metabolic processes.

A heatmap analysis of the top 15 orthologous genes in the first-generation treatment with methanol as a co-metabolic substrate was performed, utilizing the Silva database 16S phylogeny, Tax4Fun, PICRUSt conversion, and KEGG functional annotation of 16S RNA gene sequences. Metabolic differences between the WH4 group and other samples were observed. WH4 did not exhibit a higher relative abundance for any k0 compared to other samples, and the relative abundance of other metabolic pathways was also low. Only k02051, k02049, and k02050 showed relatively higher abundance, but these three metabolic pathways were also present at relatively high abundance in other samples. These pathways are related to amino acid transport [43] but are not exclusive to WH4, as the relative abundance of such proteins was also high in WH2. Additionally, the relative abundance of other proteins in WH4, when compared with those in other samples, is comparatively lower. This includes k01995-k01999, a cluster of proteins associated with branched-chain amino acid transport [44], as well as k03088 and k00799. K03088 represents a class of RNA polymerases [45], while k00799 represents glutathione S-transferase, involved in the oxidative breakdown of ingested sugars within cells [46]. The lower abundance of k00799 may suggest that WH4 is more likely to utilize carbon sources other than glucose, such as PFOS or methanol.

In order to further investigate potential functional variations between the WH4 consortium and the individual Hyphomicrobium bacterium, we conducted an enrichment analysis utilizing the complete genome sequence of Hyphomicrobium sp. strain MC1, a recognized species capable of chloromethane degradation (Fig 7c). The y-axis in this figure represents the relative abundance or proportion of the selected KEGG pathways within the MC1 genome. This comparative evaluation allows us to assess the enrichment or depletion of these pathways in the reference Hyphomicrobium strain, thereby facilitating the identification of potential functional disparities between the individual bacterium and the WH4 microbial consortium. A chloromethane-degrading bacterium, Hyphomicrobium sp. Strain MC1, with the complete genomic sequence deposited in the GenBank/EMBL database under the accession number FQ859181 [47], was selected. An enrichment analysis was conducted on MC1’s complete genome based on the KEGG database, selecting several pathways with relatively high proportions for presentation. It was found that in MC1, k02051 and k02049 have relatively high proportions, similar to the WH5 bacterial community, whereas k02050 is comparatively low in proportion. K02051 and k02049 are ATP-binding protein and substrate-binding protein of the NitT/TauT family transport system, respectively, while k02050 is a permease of the transport system [43]. This suggests subtle differences between the microbial consortium and individual bacteria in some amino acid transport pathways. Although no differences were found in terms of dehalogenation, these slight discrepancies in metabolic pathways might contribute to the functional differences between the consortium and individual bacteria.

The 25 most abundant COG annotations across distinct sample points were identified. In WH2, 23 functional groups exhibited a comparatively higher abundance. In contrast, the abundance of these 23 functional groups was lower in WH4, while the remaining 2 functional groups, associated with cell skeleton and RNA processing and modification, showed higher relative abundance. This phenomenon may be attributed to the toxic effects of fluorinated substances that constrain cell growth [48]. Notably, the relative abundance of functional groups related to lipid and carbohydrate transport was lower in WH4 than in other treatments, indicating the possibility of alternative nutrient transport mechanisms in WH4. Nonetheless, PFOS toxicity adversely affects diverse cellular functions.

## 4. Conclusion

The challenge of biodegrading recalcitrant pollutants such as PFOS is significant due to their chemical stability and the limited discovery of effective degrading microorganisms. Relying solely on isolated bacterial strains is problematic due to gaps in individual metabolic capabilities and their instability under changing conditions. In this study, an attempt was made to address these issues through a top-down functional screening of synthetic microbial consortia enriched from long-term PFOS contaminated soil.

Insights provided by our findings highlight the potential of microbial partnerships in overcoming the intractability of pollutants. A consortium primarily composed of Hyphomicrobium (46.7%) and uncultivated microorganisms (53.0%) was identified, demonstrating the ability to reduce PFOS concentration by 56.7% over 20 days upon methanol supplementation. This microbial partnership exhibited remarkable resilience under sustained PFOS stress, persisting over two generations.

Detailed analyses of the composition and predicted functions of this consortium offer novel perspectives. Although no specific dehalogenation genes were identified, the WH4 group displayed distinct metabolic profiles compared to other treatments and individual Hyphomicrobium, with lower representation of pathways involved in branched amino acid and RNA transport. Subtle discrepancies in certain amino acid transporters within the consortium, when compared to individual Hyphomicrobium metabolism, may partially account for the observed differences in degradation activities.

These findings suggest that the unclassified community members play a supportive metabolic role, complementing the inherent PFOS degradation capacity of Hyphomicrobium. Methanol supplementation facilitated core growth processes by mitigating toxicity, temporarily enhancing the degradation proficiency of this partnership. However, the preferential utilization of methanol eventually compromised stability upon serial transfer, highlighting the need for optimization of cultivation strategies.

The limitations associated with using functional prediction tools like PICRUSt on microbial communities where a substantial portion of the members remain unclassified should be acknowledged. The availability of reference genome data, which is often lacking for uncultured and uncharacterized microorganisms, heavily influences these in silico approaches. In the case of the WH4 consortium, where 53.0% of the members were unclassified at the species or genus level, caution should be exercised when interpreting the functional predictions derived from 16S rRNA gene sequences. The aim of the PICRUSt analysis performed in this study was to provide insights into the potential metabolic capabilities of the WH4 consortium, but it may not accurately represent the full functional potential of the community. The unclassified members, constituting a significant proportion, likely harbor novel and uncharted metabolic pathways that are not captured by the reference genomes utilized by PICRUSt. Furthermore, the individual-based functional predictions may not adequately reflect the complex interspecies interactions and synergistic metabolic activities within the consortium.

To overcome these limitations, alternative approaches such as metagenomics and metatranscriptomics should be explored in future studies. These techniques can provide a more comprehensive and unbiased assessment of the functional potential and gene expression profiles within microbial consortia. By employing these methods, a better understanding of the actual metabolic capabilities contributing to the observed PFOS degradation, including the roles of the unclassified members, can be achieved.

Therefore, this study emphasizes the potential of synthetic microbial ecology approaches in unlocking the decomposition abilities of otherwise inaccessible pollutants. Understanding consortia capable of attenuating pollutants paves the way for optimized bioremediation strategies targeting persistent contaminants like PFOS. Future studies that harness characterized consortia through domestication regimes have the potential to unleash their full degradation capabilities at larger scales. Addressing ecological challenges through interspecies metabolic cooperation showcases the broad importance and practical applications of this field.
